# Clinical characteristics and pharmacological treatment of new daily persistent headache (NDPH)

**DOI:** 10.1186/1129-2377-14-S1-P62

**Published:** 2013-02-21

**Authors:** H Tachibana, D Danno

**Affiliations:** 1 Hyogo College of Medicine, Japan

## Introduction

Despite the fact that NDPH has been considered one of the most refractory headaches, there are limited data available regarding the clinical characteristics, pharmacological treatment and prognosis of NDPH.

## Purpose

To discuss cases which were identified as NDPH by the International Headache Society diagnostic criteria.

## Methods

Our NDPH patients consisted of 8 females and 3 males, with an onset age ranging from 15 to 72 years old. The duration of the headaches, location, intensity and nature of the pain, precipitating factors and treatments were investigated.

## Results

The duration of the headaches ranged from 9 months to 24 years. Pain was diffuse in 3 patients, occipital-neck area in 4, frontal in 1 and left-sided in 1. Pain intensity was mild in 1 case, mild to moderate in 2, moderate in 7 and moderate to severe in 1. The nature of the pain was more similar to a chronic tension-type headache (73%) than a migraine-type, although 4 cases were found to be throbbing in nature. Headaches were related to stressful life events in 2 patients and exercise in 1 case. While two of the patients had received stellate ganglion blocks, all patients had received several kinds of drugs including analgesics, muscle relaxants, tricyclic antidepressants, selective serotonin reuptake inhibitors, anti-anxiety agents and anti-epileptic drugs. Nine patients were refractory to the pharmacological treatments.

## Conclusion

Despite the general tendency that the pain of NDPH is dull, patients occasionally suffer from bouts of throbbing pain. NDPH is the refractory headache and requires optimal pharmacological regimens on an individual basis.

## Competing interests

None
